# A potential link between polycystic ovary syndrome and non-alcoholic fatty liver disease: an update meta-analysis

**DOI:** 10.1186/s12978-018-0519-2

**Published:** 2018-05-10

**Authors:** Jia Wu, Xin-Yu Yao, Ru-Xia Shi, Su-Fen Liu, Xiao-Yong Wang

**Affiliations:** 1 Department of Gynecology, Changzhou No. 2 Hospital, Affiliated with Nanjing Medical University, Changzhou, 213000 China; 2 Department of Gastroenterology, Changzhou No. 2 Hospital, Affiliated with Nanjing Medical University, Changzhou, 213000 China

**Keywords:** Polycystic ovary syndrome, Non-alcoholic fatty liver disease, Obesity, Hyperandrogenism, Meta-analysis

## Abstract

**Background:**

Epidemiological literature regarding the effect of polycystic ovary syndrome (PCOS) as a risk factor for non-alcoholic fatty liver disease (NAFLD) remains inconsistent. Furthermore, it remains debatable whether NAFLD is associated with PCOS as a consequence of shared risk factors or whether PCOS contributes to NAFLD in an independent fashion. Therefore, this meta-analysis was conducted.

**Methods:**

This meta-analysis was conducted in accordance with the guidelines of the Preferred Reporting Items for Systematic Reviews and Meta-Analyses (PRISMA). Relevant studies published before May 2017 were identified and retrieved from PubMed and Web of Science databases. The data were extracted, and the pooled odds ratios (ORs) and 95% confidence intervals (95% CIs) were calculated.

**Results:**

A total of 17 studies were included into the present analysis. Compared to the control group, the risk of NAFLD in the PCOS group was higher (OR = 2.25, 95% CI = 1.95–2.60). When stratified by BMI and geographic location, the results indicated that the frequency of NAFLD risk was significantly higher in obese subjects (OR = 3.01, 95% CI = 1.88–4.82), non-obese subjects (OR = 2.07, 95% CI = 1.12–3.85), subjects from Europe (OR = 2.00, 95% CI = 1.58–2.52), subjects from the Asia-Pacific Region, (OR = 2.32, 95% CI = 1.89–2.84) and subjects from America (OR = 2.96, 95% CI = 1.93–4.55). In addition, PCOS patients with hyperandrogenism (HA) had a significantly higher risk of NAFLD, compared with controls (OR = 3.31, 95% CI = 2.58–4.24). However, there was no association between PCOS patients without HA and higher risk of NAFLD (OR = 1.46; 95% CI =0.55–3.87). The results of this meta-analysis should be interpreted with caution due to the small number of observational studies and possible confounding factors.

**Conclusion:**

The meta-analysis results suggest that PCOS is significantly associated with high risk of NAFLD. Although this association was independent of obesity and geographic region, it might be correlated with HA.

## Plain English summary

At present, a question that should be addressed is whether non-alcoholic fatty liver disease (NAFLD) is associated with polycystic ovary syndrome (PCOS) as a consequence of shared risk factors, or whether PCOS contributes to NAFLD in an independent fashion.

In the present study, a meta-analysis was performed to shed some light into this issue.

A total of 17 studies were included into the meta-analysis. PCOS was significantly associated with higher risk of NAFLD, with an approximately 2.3-fold higher chance. This association was independent of obesity and geographic region but might be correlated with hyperandrogenism.

In conclusion, the screening for NAFLD is only needed for hyperandrogenic women with PCOS, and not for all women with PCOS.

## Background

Non-alcoholic fatty liver disease (NAFLD) is an increasingly common form of chronic disorder in the Western world, and is characterized by fat accumulation in the liver, which is independent of significant alcohol consumption, the use of steatogenic medications, or hereditary disorders [1, 2]. NAFLD comprises of a wide spectrum of liver diseases that range from simple steatosis to nonalcoholic steatohepatitis (NASH), leading to cirrhosis [2]. Furthermore, NAFLD prevalence markedly increases with age [3]. The estimated prevalence of NAFLD in the general population ranges within 6.3–33%, with a median of 20% [2]. However, in obese or type-2 diabetes mellitus (T2DM) patients, the prevalence of NAFLD can increase to approximately 75% [4]. Most patients with NAFLD remain asymptomatic, although it can slowly progress to cirrhosis, thereby increasing the risk of hepatocellular carcinoma (HCC) [5]. NAFLD is also the most common cause of cryptogenic cirrhosis [6]. Given its high prevalence and the fact that NAFLD is becoming a major public health hazard, it is very important to unveil its potential risk factors, since early treatment can prevent the progression of liver disease. Present evidence strongly indicates that obesity, T2DM, unhealthy lifestyle, dyslipidemia, male gender and ethnicity are risk factors for NAFLD development [2, 3]. Since most patients with NAFLD have insulin resistance (IR), the association between NAFLD and IR has been widely accepted [7]. In fact, the role of IR in the pathogenesis of NAFLD has been thoroughly studied, and a strong association has been shown [8, 9].

Another disorder associated with IR is polycystic ovary syndrome (PCOS). PCOS is an exceptionally common endocrine disorder in premenopausal women of reproductive age. It is characterized by hyperandrogenism (HA), chronic anovulation and polycystic ovaries (PCO) through ovarian ultrasonography, when other etiologies are excluded [10, 11]. It is possible that the link between PCOS and NAFLD might be IR. Since the first association between NAFLD and PCOS was reported in 2005 [12], many subsequent studies have investigated the association between PCOS and risk of NAFLD. Some authors have reported that patients with PCOS have a higher prevalence of NAFLD than controls [13–15]. However, other studies failed to establish a relationship between PCOS and the increased incidence of NAFLD [16, 17]. Epidemiological literature regarding the effect of PCOS as a risk factor for NAFLD development remains inconsistent and inconclusive. A meta-analysis that comprised of data until June 2013 was performed by Ramezani-Binabaj et al. They reported that (1) the prevalence of NAFLD noticeably increased in women with PCOS, and (2) PCOS may be a significant risk factor for the development of NAFLD [18]. However, the meta-analysis was limited to only seven studies, and did not include stratified analyses. PCOS itself accounts for a higher risk of NAFLD. However, other specific factors such as androgen levels or body mass index (BMI) may contribute to NAFLD development in women with PCOS. Since 2013, many studies with larger sample sizes have reported information on factors such as obesity and androgen levels [13, 14].

Therefore, an update meta-analysis was conducted to investigate the association between PCOS and the risk of NAFLD development through studies published until May 2017. In order to explore whether NAFLD is associated with PCOS as a consequence of shared risk factors, or whether PCOS contributes to NAFLD in an independent fashion, stratified analyses were performed. Furthermore, factors associated with the presence of NAFLD in PCOS women were identified, and evidence of characteristics that can help identify women who have high risk of NAFLD were provided.

## Methods

This meta-analysis was conducted in accordance with the guidelines of the Preferred Reporting Items for Systematic Reviews and Meta-Analyses (PRISMA) [19]. The research question was formulated according to the PICO model. P [population]: studies that recruited female patients as participants; I [Intervention or Exposure]: PCOS diagnosis; C [comparator]: PCOS group compared with the control group; O [outcome]: NAFLD.

### Literature search

A comprehensive search was performed on PubMed and Web of Science databases for relevant published articles and abstracts on the association between PCOS and NAFLD published until May 2017. Merely studies performed in humans and those reported in the English language were considered. For PCOS, the following search terms were used: ¨PCOS, polycystic ovary syndrome, polycystic ovary¨. These terms were combined with the following terms for NAFLD: ¨NAFLD, Non-alcoholic fatty liver disease, NASH, non-alcoholic steatotic hepatitis, hepatic steatosis¨. For the association between PCOS and NAFLD risk, “risk, risk factor” was used.

Two independent reviewers (R-X.S. and J.W.) completed the initial screening process using pre-defined inclusion criteria (as detailed below). Articles were included for further review when these were original studies with primary data, involved human subjects, and were related to the topic of interest. These search results were independently reviewed by two reviewers and were compared for discrepancies. A third reviewer (X-Y.Y.) was involved to resolve any discrepancies that may occur between the two reviewers. In addition, the reference list of selected studies was manually reviewed for additional relevant publications.

### Inclusion criteria

Studies were included in the meta-analysis when the following criteria were met: case-control studies, cohort studies and cross-sectional studies; studies that compared the prevalence of NAFLD in women with PCOS with matched controls; studies that contained sufficient data for analyses; studies published in the English language. For articles with overlapping data of the same population source, only the largest report was included.

### Exclusion criteria

Articles were excluded when any of the following criteria were met: (1) review articles, meta-analyses, letters, commentaries, or case reports; (2) duplicates or continued work of previous publications; (3) studies without complete data; (4) articles that lack a control group; (5) articles not published in the English language. Studies without complete data, lack a control group and have overlapping data of the same population source were the common characteristics of the excluded papers.

### Data extraction and quality assessment

Data extracted from each study included the following: name of the first author(s), year of publication, geographic region from which the study population was derived, diagnostic criteria for PCOS and NAFLD, and the sample size. The baseline information and data were independently extracted by the two primary reviewers (X-Y.W. and J.W.) using the same standard. The results were compared, and disagreements were resolved by consensus.

The quality of the included studies was independently assessed by two primary investigators (X-Y.W. and J.W.) using the Newcastle-Ottawa Scale (NOS) [20]. Each study was assessed based on three broad perspectives: selection, comparability and exposure/outcome. The score ranged within 0–9. Discrepancies were resolved by discussion through a third author (X-Y.Y.).

### Statistical analysis

The association between the prevalence of NAFLD in women with PCOS and matched controls was calculated using odds ratios (ORs) and 95% confidence intervals (CIs). In addition to the overall comparisons, subgroup analyses were performed based on geographic region, BMI, diagnostic criteria for PCOS and NAFLD, and androgen levels from articles with available adequate data. Heterogeneity between studies was assessed by *Q*-test and *I*^*2*^-test. Heterogeneity was considered statistically significant at *P* < 0.05 or *I*^*2*^ > 50%, and a random-effects model was applied.

In the assessment for publication bias, Begg’s test [21] and Egger’s test were used [22]. If publication bias was detected, Duval and Tweedie’s trim and fill method was used to adjust the results [23]. In addition, a sensitivity analysis was performed using the leave-one-out analysis. Statistical analyses were conducted using Stata software version 12 (StataCorp, College Station, TX, USA). *P* < 0.05 was considered statistically significant.

## Results

### Summary of the literature search

**Fig. 1:**
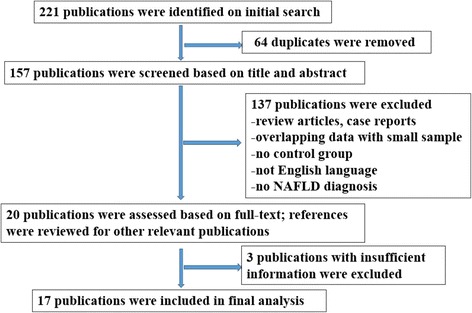
Flow diagram of the study screening and selection process

A flow chart depicting the criteria for selecting studies for the present meta-analysis is shown in Fig. 1. In the first search, a total of 221 publications were initially retrieved. Among these publications, 64 were excluded due to duplication. After reviewing the remaining 157 abstracts, 137 publications that did not meet the predefined inclusion criteria stated in the Methods section were excluded. Furthermore, three studies were excluded after reviewing the full texts due to insufficient data [24–26]. Finally, 17 studies were included for analysis [13–17, 27–38] (Table 1). The NOS scale was used to assess the quality of studies included through the two independent reviewers. The NOS results revealed that the average quality score was 5.2 (range: 4–6; Table 1).

**Table 1:** Characteristics of the studies included in the meta-analysis

First author	Year	Country	NAFLD criteria	PCOS criteria	PCOS (NAFLD)^a^	Control (NAFLD)^#^	NOS^b^
Cerda C	2007	Chile	Ultrasound	Rotterdam	41 (17)	31 (6)	5
Markou A	2010	Greece	Ultrasound	Rotterdam	17 (0)	17 (0)	6
Gutierrez-Grobe Y	2010	Mexico	Ultrasound	Not mentioned	50 (31)	147 (62)	4
Vassilatou E	2010	Greece	Ultrasound	AES	57 (21)	60 (12)	5
Hossain N	2011	USA	Histology	Rotterdam	25 (11)	25 (5)	5
Zueff LF	2012	Brazil	Ultrasound	Rotterdam	45 (33)	45 (21)	5
Karoli R	2013	India	Ultrasound	Rotterdam	54 (36)	55 (14)	5
Qu Z	2013	China	Ultrasound	Rotterdam	602 (198)	588 (109)	5
Kahal H	2014	UK	Ultrasound	Rotterdam	19 (7)	17 (0)	6
Bohdanowicz-Pawlak A	2014	Poland	Ultrasound	Rotterdam	184 (106)	125 (62)	5
Kuliczkowska Plaksej J	2014	Poland	Ultrasound	Rotterdam	173 (92)	125 (40)	5
Romanowski MD	2015	Brazil	Ultrasound	AES	101 (24)	30 (1)	5
Vassilatou E	2015	Greece	Ultrasound	Rotterdam	40 (31)	70 (40)	6
Macut D	2016	Greece Serbia	NAFLD-LFS	Rotterdam	600 (304)	125 (43)	5
Ayonrinde OT	2016	Australia	Ultrasound	NIH	32 (12)	167 (25)	5
Jie C	2017	China	Ultrasound	Rotterdam	400 (225)	100 (38)	5
Kim JJ	2017	Korea	Ultrasound	Rotterdam	275 (15)	892 (25)	6

AES: Androgen Excess Society criteria

NAFLD-LFS: NAFLD liver fat score

^a^PCOS (NAFLD) means the total number of PCOS and the number of NAFLD in PCOS group

^#^control (NAFLD) means the total number of control and the number of NAFLD in control group

^b^The quality of the included studies was assessed using the Newcastle-Ottawa Scale (NOS) and the score ranged between 0 and 9

### Data analysis

**Fig. 2:**
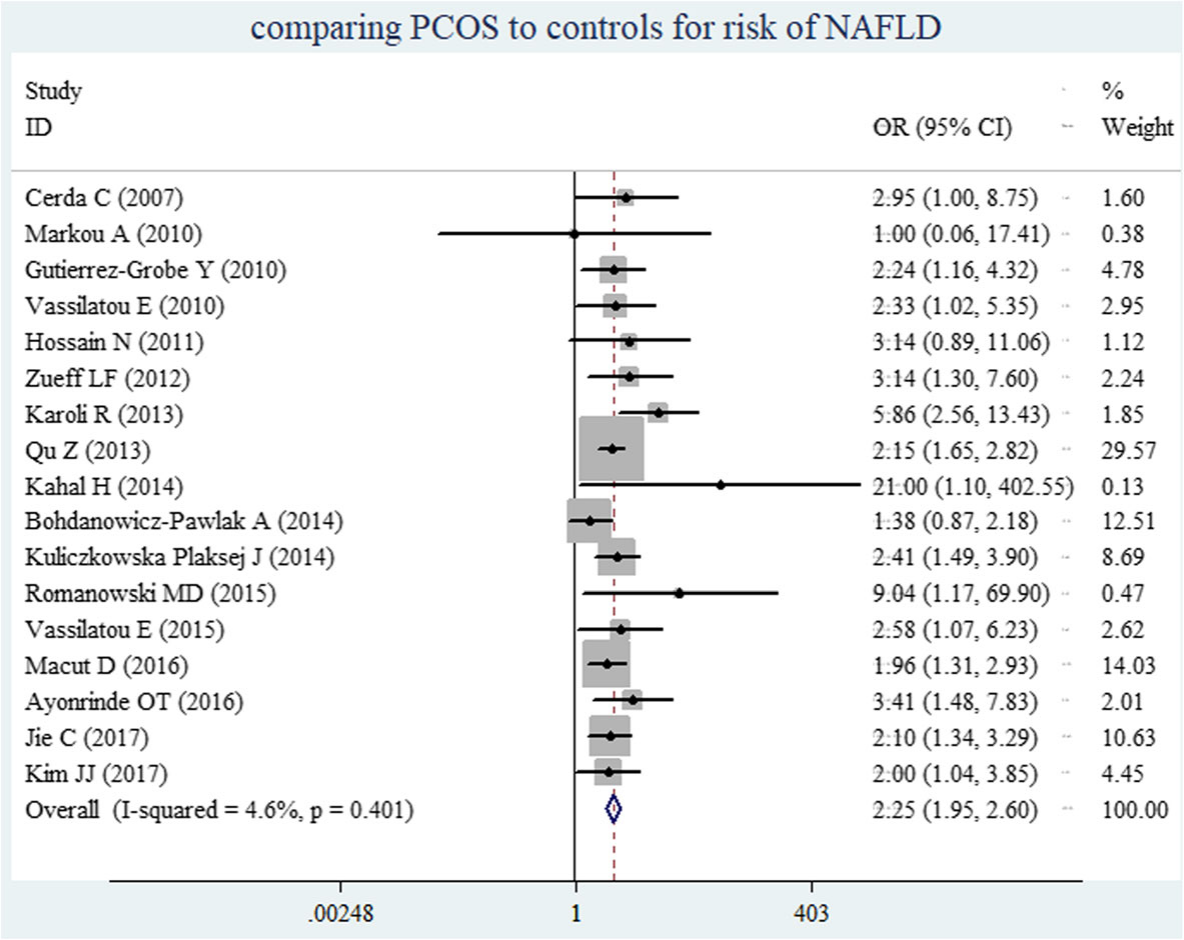
The forest plots show the individual and pooled ORs (95% CIs) obtained from studies when comparing PCOS to controls for risk of NAFLD

A total of 17 studies that compared women with PCOS with matched controls with regard to NAFLD risk were included. There was no significant heterogeneity in the included studies (*I*^*2*^ = 4.6%, *P*-heterogeneity = 0.40). Therefore, the fixed-effects model was used for the meta-analysis. Compared to the control group, the PCOS group had a significantly higher risk of NAFLD (OR = 2.25, 95% CI = 1.95–2.60) (Fig. 2).

**Fig. 3:**
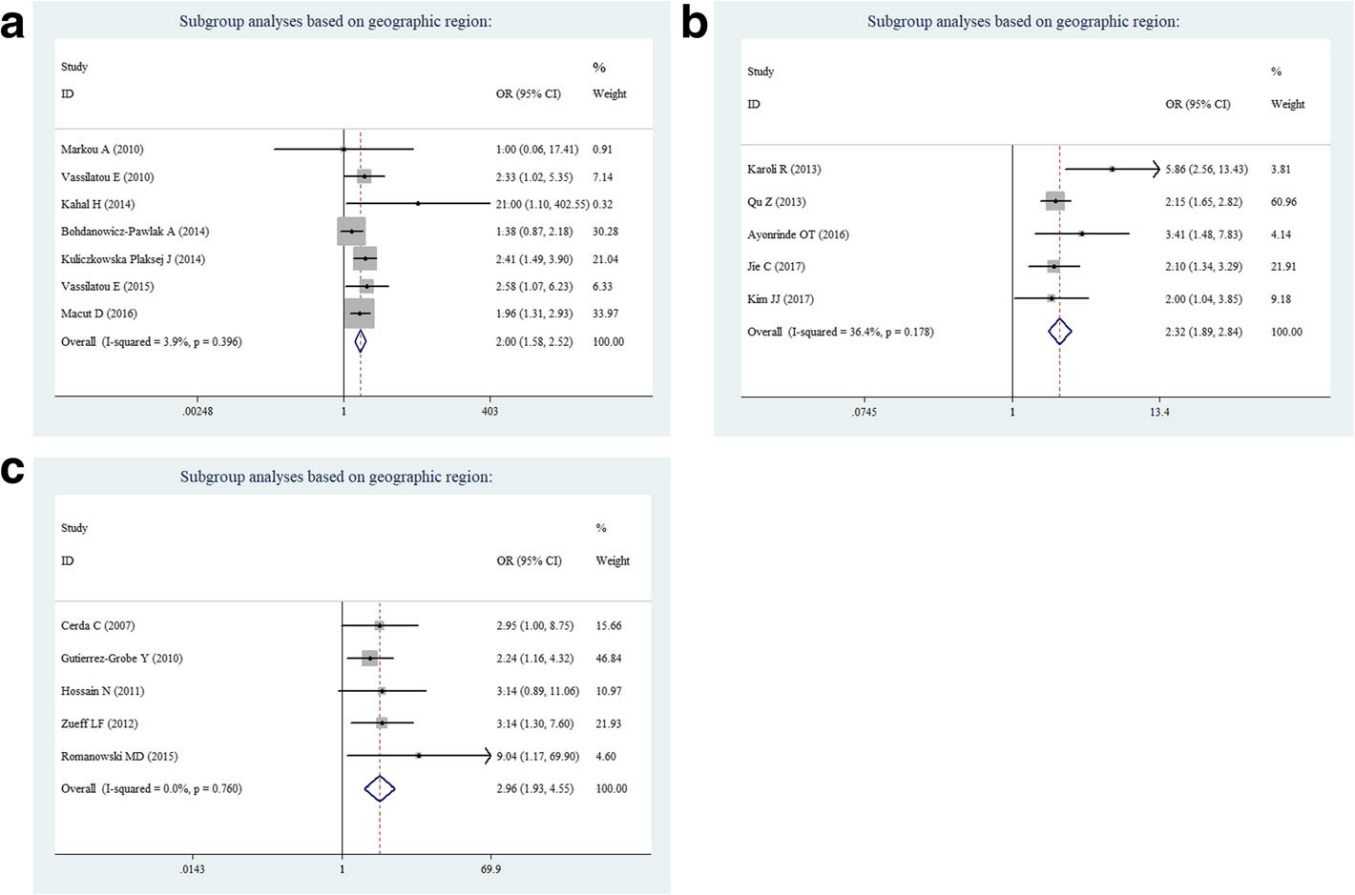
Subgroup analyses based on geographic region: **a** Europe, **b** Asia-Pacific Region, and **c** America

Subgroup analyses were also performed based on geographic region, BMI, diagnostic criteria for PCOS or NAFLD, and androgen levels. When stratified by geographic location, a significantly increased risk for developing PCOS-associated NAFLD was found in Europe (OR = 2.00, 95% CI = 1.58–2.52; Fig. 3a), the Asia-Pacific Region, (OR = 2.32, 95% CI = 1.89–2.84; Fig. 3b) and America (OR = 2.96, 95% CI = 1.93–4.55; Fig. 3c). Thus, these results strongly support that PCOS is associated with NAFLD risk, but is independent of geographic region.

**Fig. 4:**
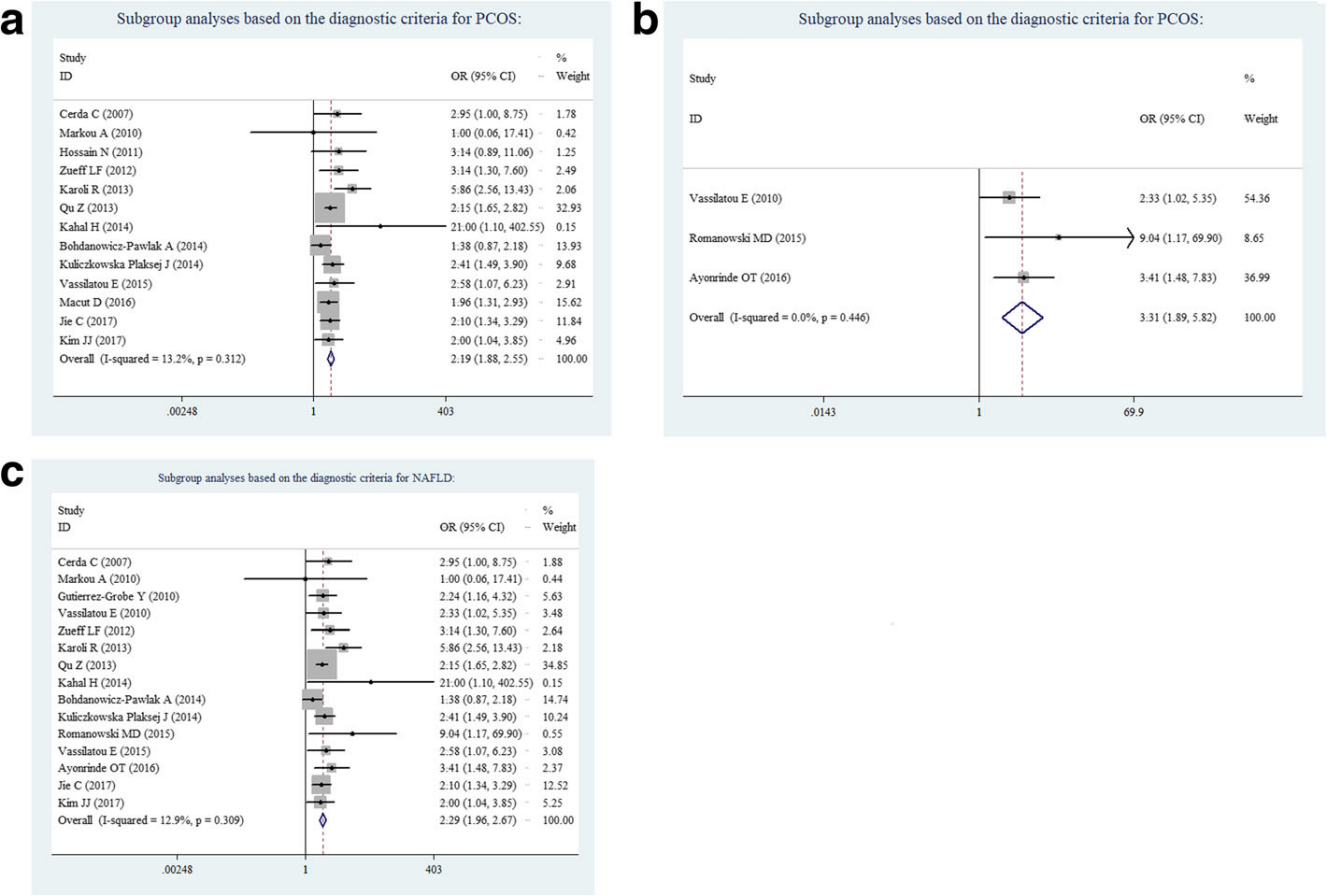
Subgroup analyses based on the diagnostic criteria for PCOS or NAFLD: **a** Rotterdam criteria, **b** no Rotterdam criteria, and **c** ultrasonography for NAFLD

According to the subgroup analyses based on the diagnostic criteria for PCOS, the effects of PCOS on NAFLD risk did not significantly vary when either the Rotterdam criteria (OR = 2.19, 95% CI = 1.88–2.55; Fig. 4a), or other criteria (OR = 3.31, 95% CI = 1.89–5.82; Fig. 4b) was used, including the National Institute of Health (NIH) criteria and the Androgen Excess Society criteria. When stratified by the diagnostic method for NAFLD using ultrasonography, it was also found that PCOS was associated with NAFLD risk (OR = 2.29, 95% CI = 1.96–2.67; Fig. 4c).

**Fig. 5:**
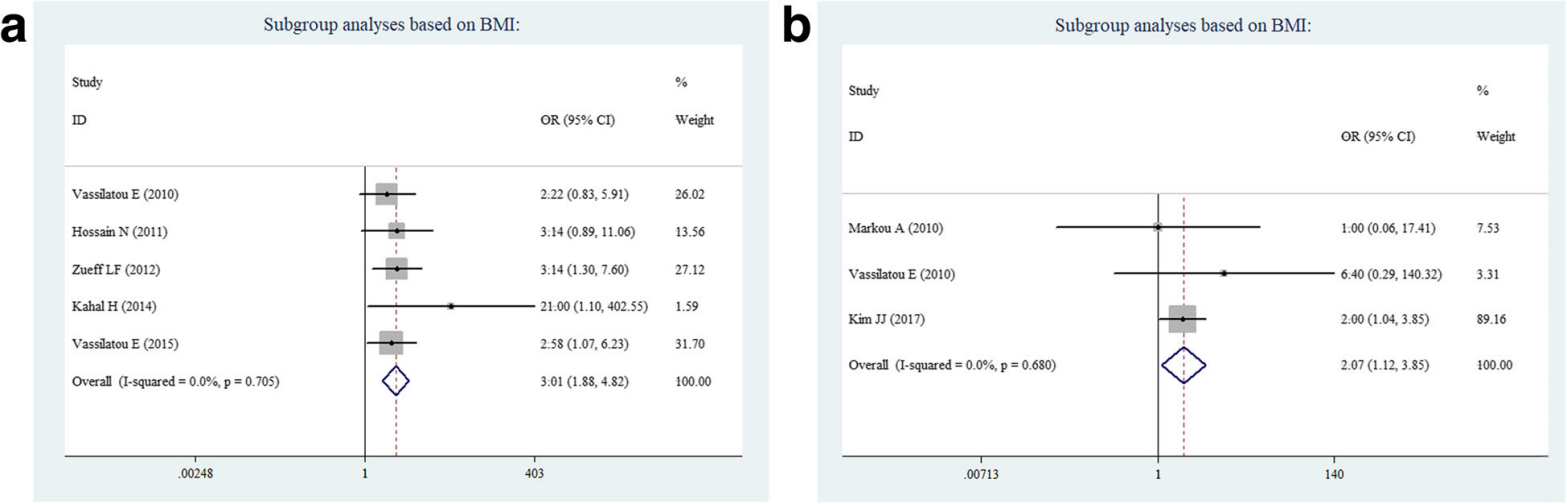
Subgroup analyses based on BMI: **a** obese subjects, and **b** non-obese subjects

When stratified by BMI, in which non-obese subjects were defined as BMI < 25 kg/m^2^, a significantly increased prevalence for NAFLD risk was found to be associated with PCOS. This was found not only in obese subjects (OR = 3.01, 95% CI = 1.88–4.82; Fig. 5a), but also in non-obese subjects (OR = 2.07, 95% CI = 1.12–3.85; Fig. 5b). Many studies have reported on the increased prevalence of NAFLD in women with PCOS, and this was most likely due to obesity and IR, as previously suggested. The present data indicates that women with PCOS have an increased prevalence of NAFLD risk, which is independent of obesity.

**Fig. 6:**
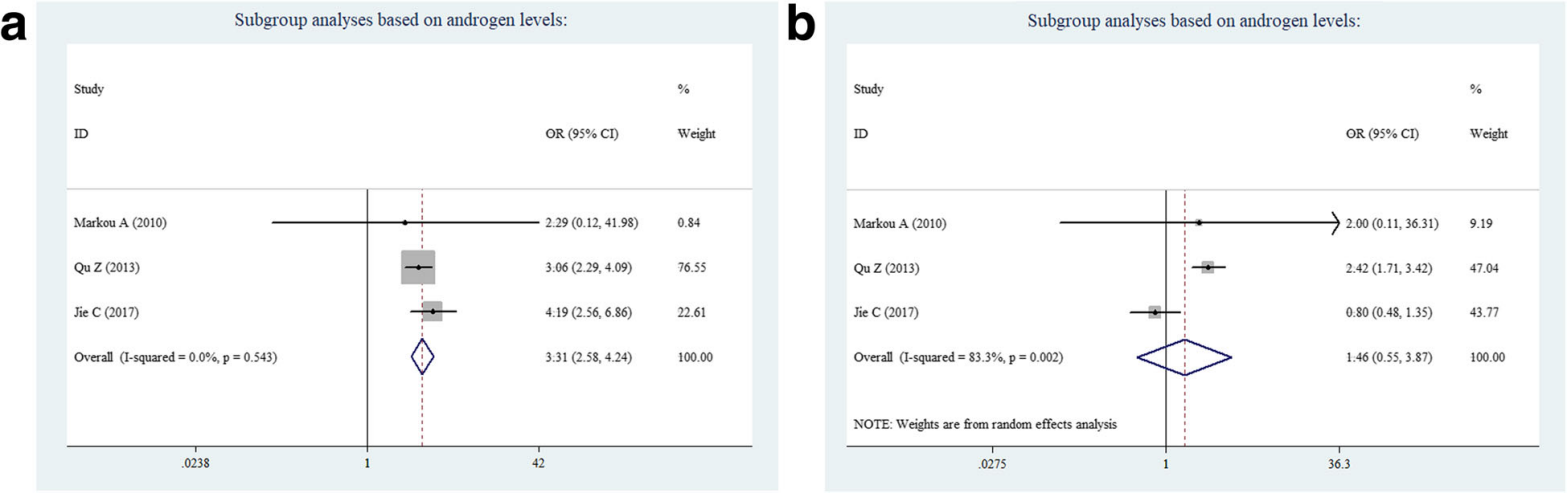
Subgroup analyses based on androgen levels: **a** HA, and **b** non-HA

According to subgroup analyses based on androgen levels, PCOS patients with HA have a significantly higher risk of NAFLD than controls (OR = 3.31; 95% CI = 2.58–4.24; Fig. 6a). However, in non-HA PCOS women, this was not associated with increased NAFLD risk when compared to controls (OR = 1.46, 95% CI =0.55–3.87; Fig. 6b). The results of the present study reveal that PCOS per se is not a risk factor that leads to the development of NAFLD, and HA is a contributing factor to the prevalence of NAFLD in women with PCOS.

Overall, no evidence of heterogeneity was found in the included studies when stratified by geographic region, BMI and diagnostic criteria for PCOS or NAFLD. However, there was significant heterogeneity in non-HA studies (*I*^*2*^ = 83.3%, P-heterogeneity = 0.002; Table 2).

**Table 2:** Stratified analyses for PCOS and risk of NAFLD

		Studies, n	OR ^a^ (95% CI)	I^2^ (%)	Heterogeneity
					0.40
Diagnostic criteria for PCOS	Rotterdam criteria	13	2.19 (1.88–2.55)	13.2	0.312
	no Rotterdam criteria^b^	3	3.31 (1.89–5.82)	0	0.446
Diagnosis for NAFLD	ultrasonography	15	2.29 (1.96–2.67)	12.9	0.309
BMI	Non-obese subjects^c^	3	2.07 (1.12–3.85)	0	0.680
	obese subjects	5	3.01 (1.88–4.82)	0	0.705
Androgen levels	HA	3	3.31 (2.58–4.24)	0	0.543
	Non- HA	3	1.46 (0.55–3.87)	83.3	0.002
Geographic region	Asia-Pacific	5	2.32 (1.89–2.84)	36.4	0.178
	America	5	2.96 (1.93–4.55)	0	0.760
	Europe	7	2.00 (1.58–2.52)	3.9	0.396

^a^OR comparing groups of PCOS to the matched controls with regard to the risk of NAFLD

^b^including NIH criteria or Androgen Excess Society criteria

^c^defined as BMI < 25 kg/m^2^

### Sensitivity analysis and publication bias

Begg’s test and Egger’s test were used to assess for publication bias for the included studies. There was evidence of publication bias in the overall meta-analysis for risk of NAFLD (Begg’s test: *P* = 0.01; Egger’s test: *P* = 0.02). Furthermore, the trim-and-fill method developed by Duval and Tweedie was used to adjust for bias [23]. The adjusted result from the fixed model had an OR of 2.08 (95% CI = 1.81–2.40), which was similar to the results of the present study (OR = 2.25, 95% CI = 1.95–2.60). Thus, the results of the present study are reliable. A sensitivity analysis was further performed by removing one study at a time from the meta-analysis. None of the results were significantly altered when a single study was removed. The pooled OR ranged within 2.18–2.38, confirming the robustness of these results.

## Discussion

At present, epidemiological literature regarding the effect of PCOS as a risk factor for NAFLD development remains inconsistent and inconclusive. This controversy needs to be clarified, since the relationship between PCOS and NAFLD is extremely relevant in clinic, both disorders are common, and the coexistence might synergistically increase the risk for both T2DM and cardiovascular disease.

In order to shed some light, a meta-analysis that included seven studies was conducted. It was reported that the prevalence of NAFLD is markedly increased in women with PCOS. Therefore, PCOS has emerged as a significant risk factor for the development of NAFLD [18]. However, a question that should be addressed is whether NAFLD is associated with PCOS as a consequence of shared risk factors, or whether PCOS contributes to NAFLD in an independent fashion. Therefore, the present meta-analysis was conducted to shed light into the relationship between PCOS and NAFLD. According to the results of the present meta-analysis, women with PCOS had a 2-fold higher chance of developing NAFLD, which was independent of BMI and geographic region. Furthermore, hyperandrogenic women with PCOS had a significantly higher risk of NAFLD than controls. However, non-HA PCOS patients were not associated with the increased prevalence of NAFLD, when compared to controls. This finding suggests that the putative mechanism linking PCOS and NAFLD is most likely associated with HA.

The association between hyperandrogenic women with PCOS and NAFLD risk is supported by several biological mechanisms. First, androgens can suppress the transcription of the low-density lipoprotein receptor (LDLR) gene, thereby prolonging the half-life of very low-density lipoprotein (VLDL) and LDL. As a result, lipids accumulate in the liver, and hyperandrogenic women with PCOS become more prone to fatty liver [39]. Second, hyperandrogenemia can induce a low-grade inflammatory state by increasing the transcripts of the androgen receptor and the release of TNF-α from mononuclear cells [40], and both contributes to the development of NAFLD [41]. Third, the pathogenesis of NAFLD is multifactorial, but IR appears to be a pivotal contributing factor [7]. Both clinical observations and animal studies have demonstrated that IR could be induced by hyperandrogenemia [42]. Su et al. reported that adult female mice treated with testosterone exhibited IR via the blockade of insulin signal transduction, without influencing body weight and body fat content [43]. Another study conducted by Polderman et al. reported that the testosterone administration to females can induce IR in healthy subjects [44]. Finally, increased androgen levels can promote visceral fat accumulation by inhibiting adenosine monophosphate-activated protein kinase (AMPK) activation, which is a potent inhibitor of lipogenesis in adipose tissues [45]. Visceral obesity is also associated with NAFLD. Thus, an excess in androgen levels may contribute to NAFLD in PCOS patients, affecting the liver directly or indirectly through the modulation of insulin sensitivity, increasing visceral adiposity, or a combination of both.

There were several limitations in the present meta-analysis. First, although the collected publications were all eligible, the studies included in the meta-analysis were limited. Meanwhile, these limited number of studies precluded any meaningful subgroup analyses stratified by important confounding factors such as IR. Second, the method used to assess NAFLD in the majority of studies was ultrasonography, and it has been demonstrated that liver biopsy is the gold standard for the diagnosis of NAFLD. The advantages of ultrasonography include non-invasiveness, reproducibility, low cost, and satisfactory sensitivity and specificity for epidemiologic studies. Third, publication bias existed among the involved studies. However, the trim and fill method was subsequently performed. The OR after adjustment changed to 2.08, which was almost consistent with the unadjusted result. Thus, the present results are reliable. Fourth, it was found that PCOS without HA was not associated with increased NAFLD risk, when compared to controls. However, few of the included studies had high heterogeneity. Finally, the entire body of evidence was observational, which may be biased due to unmeasured confounders. Although some studies were adjusted for important confounding factors, other modifiable factors were not accounted for in all these studies, such as family history, differences in diet, and/or physical activity patterns. Therefore, residual confounders may be present, which may influence these results. Thus, these observed effects may be attributable to these factors, as opposed to PCOS alone. Conversely, it could not be excluded that confounding factors may mask a more influential factor, leading to the underestimation of the effects of PCOS. Thus, future prospective high-quality studies may be necessary to conclusively address this point.

## Conclusion

In summary, the results of the meta-analysis suggest that PCOS is significantly associated with high risk of NAFLD. This association was dependent of androgen levels, which is the main feature of PCOS, and was interrelated to IR. These findings have clinical implications for NAFLD screening in hyperandrogenic women with PCOS.

## Abbreviations

AES: Androgen Excess Society criteria
CIs: Confidence intervals
HA: Hyperandrogenism
LDLR: Low-density lipoprotein receptor
MS: Metabolic syndrome
NAFLD: Non-alcoholic fatty liver disease
NASH: Nonalcoholic steatohepatitis
ORs: Odds ratios
PCOS: Polycystic ovary syndrome
T2DM: Type-2 diabetes mellitus
